# Associations between COVID-19 Incidence Rates and the Exposure to PM2.5 and NO_2_: A Nationwide Observational Study in Italy

**DOI:** 10.3390/ijerph17249318

**Published:** 2020-12-13

**Authors:** Fabiana Fiasca, Mauro Minelli, Dominga Maio, Martina Minelli, Ilaria Vergallo, Stefano Necozione, Antonella Mattei

**Affiliations:** 1 Department of Life, Health & Environmental Sciences, University of L’Aquila, 67100 L’Aquila, Italy; fabiana.fiasca@alice.it (F.F.); stefano.necozione@univaq.it (S.N.); 2 Specialistic Allergic Unit & Immunological Pathologies, PoliSmail Network, 73100 Lecce, Italy; mauro.minelli@unipegaso.it (M.M.); dmaio@polismail.it (D.M.); martinaminelli@polismail.it (M.M.); ilaria.vergallo@gmail.com (I.V.); 3 Centro Direzionale Isola F2, Pegaso Online University, 80132 Naples, Italy

**Keywords:** COVID-19, air pollution, NO_2_, PM2.5, incidence rates, epidemiology, environmental, old age index, population density, linear regression

## Abstract

The COVID-19 outbreak disproportionately affected the elderly and areas with higher population density. Among the multiple factors possibly involved, a role for air pollution has also been hypothesized. This nationwide observational study demonstrated the significant positive relationship between COVID-19 incidence rates and PM2.5 and NO_2_ levels in Italy, both considering the period 2016–2020 and the months of the epidemic, through univariate regression models, after logarithmic transformation of the variables, as the data were not normally distributed. That relationship was confirmed by a multivariate analysis showing the combined effect of the two pollutants, adjusted for the old-age index and population density. An increase in PM2.5 and NO_2_ concentrations by one unit (1 µg/m^3^) corresponded to an increase in incidence rates of 1.56 and 1.24 × 10^4^ people, respectively, taking into account the average levels of air pollutants in the period 2016–2020, and 2.79 and 1.24 × 10^4^ people during March–May 2020. Considering the entire epidemic period (March–October 2020), these increases were 1.05 and 1.01 × 10^4^ people, respectively, and could explain 59% of the variance in COVID-19 incidence rates (R^2^ = 0.59). This evidence could support the implementation of targeted responses by focusing on areas with low air quality to mitigate the spread of the disease.

## 1. Introduction

SARS-Cov-2 (Severe Acute Respiratory Syndrome-Coronavirus-2) is a new strain of coronavirus that was previously never identified in humans. The disease caused by it was referred to as “COVID-19” (where “CO” stands for corona, “VI” for virus, “D” for disease, and “19” indicates 2019, the year in which it occurred) [1]. In December 2019, the COVID-19 infection was rapidly spreading, leading to health emergencies worldwide [2,3,4,5].

It is known that in cases of viral infections, severe air pollution can have negative effects: infected people may be more susceptible to disease due to reduced body immunity, making microorganisms more invasive [6]. In a relatively recent study, conducted in Lombardy [7], some pollutants were found to be associated with an increased risk of complications and hospitalization for respiratory syncytial virus bronchiolitis (RSV).

Consequently, it is reasonable to assume that inhaled pollutants may have a severe impact of respiratory viruses on the respiratory system.

Based on this assumption, the potential association between air pollution and the COVID-19 epidemic has recently been described.

It is assumed that SARS-Cov-2 infection causes severe lung disease induced by the combined effect of PM2.5 and NO_2_ (two of the most common traffic-related air pollutants, known as TRAPs) [8]. The oxidative stress induced by PM2.5 can produce pulmonary lesions in mice; the more severe lesions were associated with altered levels of angiotensin II conversion enzyme (ACE2) expression in lung tissue. SARS-Cov-2 spike protein interacts with ACE2 on host airway cells to infect them. ACE2 is over expressed in the lower respiratory tract and allows the internalization of the virus and consequently triggers the entire pathological process that characterizes the clinical picture of COVID-19 [9,10]. The over expression of ACE2 may increase the viral load in patients exposed to pollutants, in turn depleting ACE-2 receptors and compromising host defenses. High atmospheric NO_2_ can provide a second hit causing a severe form of SARS-CoV-2 in ACE-2-depleted lungs resulting in a worse outcome [8].

Several Italian studies have already investigated the relationship between air quality and COVID-19. Fattorini et al. have shown a significant correlation between confirmed cases of COVID-19 in up to 71 Italian provinces (updated 27 April 2020) and the corresponding average concentrations in the last four years of NO_2_, PM2.5, and PM10 [11]. Bontempi et al. analyzed the relationship between the daily confirmed infection cases that occurred in March 2020 in Lombardy and Piedmont cities and corresponding daily PM10 concentration values [6]. Conticini et al. supposed that communities living in polluted areas, such as Lombardy and Emilia Romagna, might be more predisposed to die of COVID-19 because of their health status [12]. Nevertheless, in addition to the determinants of the relation under examination, it is also necessary to consider the main confounders, such as the demographic characteristics of the Italian population [13]. Scientific evidence has shown that most cases occur among the elderly and in areas with higher population density, the density being important for the spreading of coronavirus [14]. The demographic characteristics of the Italian population differ from the other countries with the lowest incidence, since the quote of the elderly is very high (on 1 January 2019, 23% of Italian population was 65 years or older). This could explain the higher incidence of COVID-19 cases in Italy, being individuals aged 70 years or older (37.6% of cases) and male patients with multiple comorbidities the most susceptible population group [15].

In order to estimate the relationship between COVID-19 and exposure to air pollutants, which also takes into account the demographic characteristics of the Italian population, given that these can significantly influence the spread and the susceptibility to infection, a nationwide observational study was conducted with the following basic assumption: COVID-19 infection has affected the population most exposed to air pollutants and increases where people are older, and the population density is higher.

## 2. Materials and Methods

This retrospective observational study was conducted using three distinct informational flows of free consultation.

1. The European Environment Agency website was used to extrapolate the average weekly levels of PM2.5 and NO_2_, stratified by provinces and metropolitan cities [16]. Data on PM2.5 and NO_2_ concentrations were available for 62 and 67 provinces out of a total of 110, respectively. Three periods were considered: 2016–2020 years, to evaluate historical data, March–May 2020, to assess current concentrations during the months of the first wave of coronavirus, and March-October 2020, to analyze pollutant levels for the entire epidemic period. The average concentrations for the periods under review were calculated and expressed as mean with a 95% confidence interval (95% CI).
2. The website of the Department of Civil Protection was checked in order to extract the number of COVID-19 cases, stratified by provinces, updated on 24 June 2020, for the first wave of coronavirus infection, and on 3 November 2020, to consider the entire epidemic period [17]. Data were released every day at 6 PM (UTC +1 h) and archived on GitHub.
3. The national database of the Italian Institute of Statistics (ISTAT) was used to extract the annual resident population on 1 January 2019, for the calculation of COVID-19 incidence rates expressed for 10,000 people, also stratified by provinces [18]. Confidence intervals of 95% were reported for the overall incidence rates.

The data provided by the Department of Civil Protection websites did not contain any patient identifiers and were therefore completely anonymous. Consequently, notification of the study to Ethics Committees was not applicable, nor was informed consent of patients required, and the research was completed in accordance with the Helsinki Declaration.

The Shapiro–Wilk test was used to evaluate the normality of the residuals [19]. The hypothesis was that the residuals were normally distributed (null hypothesis) versus the idea that the residuals were not normally distributed (alternative hypothesis). As the data were not normally distributed, a logarithmic transformation was performed. A linear regression model was used to examine the association between PM2.5 and NO_2_ levels and COVID-19 incidence rates in the Italian provinces. The variables statistically associated with COVID-19 incidence rates in univariate linear regression models were included in the multivariate linear regression analysis, considering old-age index ((Population ≥ 65 years old/Population ≤ 14 years old) × 100) and population density (people per square kilometer) as potential confounders.

Statistical significance was set at *p* < 0.05. The data were processed using the STATA/IC 15.0 statistical package (Stata Corp LP, College Station, TX, USA).

## 3. Results

The European Environment Agency reported an average pollutant concentration for PM2.5 and NO_2_ of 16.84 (15.61–18.07) and 27.97 (26.10–29.83), respectively, between 2016 and 2020. Considering the first months of the epidemic (March-May 2020), the mean values decreased to 12.94 (12.05–13.84) and 15.18 (13.75–16.61), respectively. These values were, respectively, 11.32 (10.55–12.09) and 16.94 (15.30–18.58), considering the period from March to October 2020.

The Civil Protection Department registered 239,410 COVID-19 cases in Italy until 24 June 2020, with an overall incidence rate of 39.25 × 10,000 people (32.02–46.48), and 743,149 COVID-19 cases until 3 November 2020, with an overall incidence rate of 116.03 × 10,000 people (104.11–127.95). As shown in Table 1, among the provinces for which both data on pollutants and COVID-19 were available, during the first months of epidemic, the lowest incidence of infection was found for Palermo (3.98 × 10^4^ people), almost 50 times lower than that of Cremona, which recorded the highest infection level (187.02 × 10^4^ people). Considering the updating until 3 November, the lowest incidence rate was found for Lecce (18.80 × 10^4^ people), almost 14 times lower than that of the province of Piacenza, which recorded the highest level of infection (257.66 × 10^4^ people). Analyzing the characteristics of the Italian population, Biella was the province with the highest old-age index (268%), while the highest population density was found in Naples (2617 people/km^2^).

The relationship between air pollutant concentrations and COVID-19 incidence rates was analyzed through a linear regression model (Table 2). As the data were not normally distributed, a logarithmic transformation of all variables introduced in the model was performed (*p* = 0.273 using the Shapiro–Wilk test). The univariate regression model demonstrated a significant positive relationship between PM2.5 and NO_2_ levels, considering both the period 2016–2020 (PM2.5: β coefficient = 1.99; 95% CI = 1.28–2.69; *p* < 0.001. NO_2_: β coefficient = 1.63; 95% CI = 0.83–2.44; *p* < 0.001), the months of the first epidemic peak (PM2.5: β coefficient = 3.26; 95% CI = 1.93–4.60; *p* < 0.001. NO_2_: β coefficient = 1.36; 95% CI = 0.80–1.91; *p* < 0.001) and the months from March to October 2020 (PM2.5: β coefficient = 1.07; 95% CI = 0.07–2.06; *p* = 0.037. NO_2_: β coefficient = 0.83; 95% CI = 0.51–1.16; *p* < 0.001). Considering the combined effect of the two pollutants, corrected for old-age index and population density, an increase in PM2.5 and NO_2_ concentrations by one unit (1 µg/m^3^) corresponded to an increase of COVID-19 incidence rates of 1.56 (0.83–2.29) and 1.24 (0.40–2.07) × 10^4^ people, respectively, based on the levels of air pollutant in the period 2016–2020. An adjusted R squared value of 0.51 indicated that 51% of the variance of the COVID-19 incidence rate was explained by the independent variables. This value rose to 59%, considering the entire epidemic period (March–October 2020), and to 68% considering the concentrations of air pollutant during the first three epidemic months (March–May 2020).

## 4. Discussion

The COVID-19 pandemic has social and economic repercussions; therefore, epidemiological evidence is needed to identify potential vulnerability factors to help targeted responses. Among proposed vulnerability factors, air pollution has received particular attention in the last few months, as acute exposure can affect respiratory health, causing pulmonary inflammation that can reduce lung function through bronchoconstriction or an alteration of the pulmonary immune system; chronic exposure exacerbates inflammation, cellular proliferation, extracellular matrix reorganization and weakens pulmonary immune response [20]. It is also important to assess the effect of exposure to air pollution and other social determinants of health. The overall distribution of the elderly population in Italy and in the provinces with higher population density led us to analyze the relationship between exposure to air pollutant concentrations, PM2.5 and NO_2_, and COVID-19 incidence rates adjusted to old-age index and population density of the provinces. The classical epidemiological approach of the effects of outdoor air pollution on health distinguishes between short and long-term exposure. The long-term effects increase the size of the susceptible population. At the same time, in the short term, there may be an increase in the rate of propagation or a complication of the clinical conditions of patients already suffering from concomitant diseases [13]. We considered the mean concentrations of pollutants in the 2016–2020 timeframe for long exposure and the average concentrations during the epidemic months, March–May 2020, for short exposure.

Looking at the independent effects of the two pollutants in the period 2016–2020, the univariate regression model showed a significant positive relationship between PM2.5 and NO_2_ concentrations and COVID-19 incidence rates. The relationship was confirmed after a multivariate analysis showing the combined effect of the two pollutants, corrected for old-age index and population density. In quantitative terms, we can say that an increase of one unit of pollutant concentrations (1 µg/m^3^) corresponded to an increase of COVID-19 incidence rates of 1.56 (0.83–2.29) and 1.24 (0.40–2.27) × 10,000 people, respectively. The multivariate model showed that the coefficient of determination increased to 0.51. This value indicated that 51% of the variance of COVID-19 incidence rate was explained by the independent variables.

A previous study by Fattorini et al. found that long-term data on air-quality are significantly correlated with cases of COVID-19 in up to 71 Italian provinces, providing further evidence that chronic exposure to air contamination may represent a favorable context for the spread of the virus [11]. In line with the suggestions provided by Ancona C and Wu X [13,21], our study added the adjustment for two potential confounding factors, old-age index and population density, that could positively influence the spread of the disease.

Considering the months of the first epidemic peak, the univariate regression model showed a significant positive relationship between PM2.5 and NO_2_ concentrations and COVID-19 incidence rates. Considering the combined effect of the two pollutants, during months of the epidemic, an increase in pollutant concentrations by one unit (1 µg/m^3^) corresponded to an increase in COVID-19 incidence rates of 2.79 (1.74–3.83) and 1.24 (0.70–1.79) × 10^4^ people, respectively. In the multivariate model, the coefficient of determination increased to 0.68. This value indicates that 68% of the COVID-19 incidence rate variance was collectively explained by the concentration of air pollutants.

Our evidence supports the “double-hit” hypothesis of Frontera et al. [8], whose assumption was that chronic exposure to PM2.5 causes an overexpression of the ACE-2 alveolar receptor. This may increase the viral load in patients exposed to pollutants, in turn depleting ACE-2 receptors and compromising the host defenses. High atmospheric NO_2_ may provide a second hit causing a severe form of SARS-CoV-2 in ACE-2 depleted lungs, resulting in a worse outcome.

A study investigating the potential association between the use of angiotensin-receptor blockers (ARBs) and ACE inhibitors and the risk of COVID-19 showed that this use was more frequent among patients who were infected with SARS-CoV-2 than among the large number of controls who were matched by age, sex, and place of residence, providing evidence that the use of ARBSs or ACE inhibitors was independently associated with the risk of COVID-19 in patients with mild-to-moderate disease or those with severe disease [22]. Finally, it is also important to emphasize that exposure to air pollutants among the population is not random and might intersect with other social determinants of health. Several studies have confirmed that patients with COVID-19 had a higher baseline prevalence of cardiovascular conditions and diseases for which treatment with ARBs and ACE inhibitors is often used [3,20,23,24,25,26,27].

Vulnerability to COVID-19 disease seems to be increased by PM2.5, as Lin et al. hypothesized that instillation of particulate matter 2.5 induced acute lung injury and attenuated injury recovery in ACE2 knockout mice [9].

Looking at the whole period considered (March–October 2020), the role of air pollutants in explaining the variance of the COVID-19 incidence rate fell to 59%, underlining that COVID-19 epidemic is a multi-factorial phenomenon in which air pollutants play an important role. In addition, it should be remembered that since 24 June 2020, the Ministry of Health has changed the data collection system on the spread of COVID-19: positive cases were no longer indicated according to the province of notification but according to the province of residence or domicile [28]. This could partly explain the different result obtained, considering the first wave or the entire epidemic period.

There are several limitations of the study. Firstly, this is an epidemiological observational investigation. It cannot, therefore, provide the molecular mechanisms underlying the formulated hypothesis, which should be evaluated through appropriate experimental models. Moreover, the multivariate linear regression was adjusted for two potential confounding factors, old-age index and population density, but other factors such as lifestyle (e.g., diet or smoking habits), the prevalence of pre-existing conditions such as cardiovascular and respiratory problems and diabetes before the pandemic, the capacity of the healthcare system, the case identification practices (e.g., the rate of the population tested and the percentage of positive tests in relation to the total number of tests carried out), or the duration of confinement, among others, should be taken into account [20].

## 5. Conclusions

Documenting the impact of air pollution on COVID-19 incidence rates could be crucial to implement targeted responses focusing on areas with low air quality. This observational study aims to be a further enrichment of the ongoing scientific discussion, to identify potential vulnerability factors to help mitigate the spread of the disease, because it analyzed the independent and combined effect of pollutants on the incidence rate adjusted for old age index and population density, as the incidence of coronavirus diseases increases among the elderly and in areas with the highest population density. Such evidence could help politicians about which measures to take during the pandemic- or traffic-related measures in a post-COVID-19 era, as pandemic preparedness strategies for climate adaptation are imperative.

## Figures and Tables

**Table 1:** COVID-19 incidence rates × 10^4^ people, old-age index and population density stratified by Italian provinces and metropolitan cities.

Provinces	Incidence Rates (Update on 24 June 2020)	Incidence Rates (Update on 3 November 2020)	Old-Age Index (%)	Population Density (People/km^2^)
**Piedmont**				
Turin (metropolitan city)	70.71	190.38	201	331
Novara	75.91	167.91	183	275
Asti *	88.08	179.03	215	142
Biella	59.81	144.44	268	192
**Liguria**				
Savona	56.21	129.82	265	179
Genova (metropolitan city)	66.16	225.88	257	459
La Spezia	39.05	187.27	242	249
**Lombardy**				
Varese	43.71	198.57	175	743
Como	68.39	193.72	169	468
Milan (metropolitan city)	75.13	246.93	167	2,063
Bergamo	128.97	172.28	145	405
Brescia	124.11	178.66	151	265
Pavia	102.69	204.98	198	184
Cremona	187.02	254.01	189	203
Lecco	84.29	178.17	175	419
**Trentino South Tyrol**				
Bolzano/Bozen *	49.84	188.90	124	72
Trento	90.48	181.52	154	87
**Veneto**				
Verona	55.60	125.83	158	299
Vicenza	33.27	124.17	159	317
Treviso	30.15	145.65	157	358
Venice (metropolitan city)	31.52	109.97	198	345
Padova	42.23	133.95	170	437
**Friuli Venzia Giulia**				
Udine	18.89	84.20	224	106
Trieste	59.76	168.56	259	1,103
Pordenone	22.42	69.14	177	137
**Emlia Romagna**				
Piacenza	156.40	257.66	196	111
Parma	81.21	133.87	175	131
Reggio nell’Emilia	93.04	182.13	150	232
Modena	55.11	129.83	164	262
Bologna (metropolitan city)	50.81	126.20	190	274
Ferrara	29.77	84.72	256	131
Ravenna	26.28	86.35	201	209
Forlì-Cesena	43.88	114.70	184	166
Rimini	63.41	144.80	173	392
**Marche**				
Pesaro e Urbino	77.44	109.61	186	140
Ancona	39.95	95.62	195	240
**Tuscany**				
Massa-Carrara	54.17	160.96	241	169
Lucca	34.95	127.18	214	219
Florence (metropolitan city)	31.59	152.56	201	288
Livorno	14.24	110.21	228	276
Pisa	22.12	155.05	188	171
Prato	20.61	165.47	158	705
**Umbria**				
Perugia	15.38	130.48	194	104
Terni	16.65	114.40	238	106
**Lazio**				
Rome (metropolitan city)	13.40	88.99	157	810
Latina	10.48	69.72	155	255
**Campania**				
Caserta	5.24	126.88	117	348
Benevento	7.55	40.16	186	133
Naples (metropolitan city)	8.58	139.03	117	2,617
Avellino	13.21	79.44	179	149
Salerno	6.30	63.97	154	222
**Abruzzo**				
Pescara	49.95	94.64	179	259
**Molise**				
Campobasso	16.43	53.17	215	76
**Apulia**				
Foggia	18.84	79.79	155	89
Bari (metropolitan city)	11.92	70.46	163	324
Taranto	4.86	35.67	174	234
Lecce	6.54	18.80	195	284
Barletta-Andria-Trani	9.75	59.43	138	253
**Calabria**				
Cosenza	6.64	26.61	175	105
Catanzaro	5.98	25.26	169	148
Reggio Calabria (metropolitan city)	5.28	39.63	155	171
**Sicily**				
Palermo (metropolitan city)	3.98	61.62	144	250
Messina (metropolitan city) *	7.54	35.23	186	192
Catania (metropolitan city) *	7.02	58.37	135	310
Siracusa *	8.05	39.68	159	188
**Sardinia**				
Sassari	17.79	91.85	194	64
Cagliari (metropolitan city)	5.80	49.59	196	345

* no PM2.5 concentrations available.

**Table 2:** Univariate and multivariate linear regression analysis with logarithmic transformation for the associations between COVID-19 incidence rates and the explanatory variables (PM2.5 and NO_2_ average concentrations).

Air Pollutants	Mean (95% CI) µg/m^3^	Univariate Linear Regression				Multivariate Linear Regression			
		β Coefficient	95% CI	*p*-Value	R^2^	β Coefficient *	95% CI	*p*-Value	Adjusted R^2^
PM2.5 (2016–2020 years)	16.84 (15.61–18.07)	1.99	1.28–2.69	**<0.001**	0.35	1.56	0.83–2.29	**<0.001**	0.51
NO_2_ (2016–2020 years)	27.97 (26.10–29.83)	1.63	0.83–2.44	**<0.001**	0.20	1.24	0.40–2.07	**0.004**	
PM2.5 (March–May 2020)	12.94 (12.05–13.84)	3.26	1.93–4.60	**<0.001**	0.41	2.79	1.74–3.83	**<0.001**	0.68
NO_2_ (March-May 2020)	15.18 (13.75–16.61)	1.36	0.80–1.91	**<0.001**	0.29	1.24	0.70–1.79	**<0.001**	
PM2.5 (March–October 2020)	11.32 (10.55–12.09)	1.07	0.07–2.06	**0.037**	0.12	1.05	0.33–1.78	**0.006**	0.59
NO_2_ (March–October 2020)	16.94 (15.30–18.58)	0.83	0.51–1.16	**<0.001**	0.30	1.01	0.63–1.39	**<0.001**	

* linear regression coefficient adjusted for old-age index and population density; Statistically significant association are shown in bold (*p*-value < 0.05).
